# Accuracy of echocardiography and chest tomography for pulmonary hypertension screening in patients awaiting lung transplantation

**DOI:** 10.31744/einstein_journal/2021AO5710

**Published:** 2021-12-15

**Authors:** Luiza Helena Degani-Costa, João Paulo de Assis, Pedro Paulo Pisaniello Gonçalves, Fernanda Gushken, Gilberto Szarf, José Eduardo Afonso

**Affiliations:** 1 Hospital Israelita Albert Einstein São Paulo SP Brazil Hospital Israelita Albert Einstein, São Paulo, SP, Brazil.

**Keywords:** Hypertension, pulmonary, Transplantation, Echocardiography, Tomography, x-ray computed, Thorax/diagnostic imaging, Cardiac catheterization

## Abstract

**Objective:**

To examine the accuracy of a pulmonary hypertension screening strategy based on a combination of echocardiographic data and tomographic measurements (pulmonary artery diameter and pulmonary artery diameter to ascending aorta diameter ratio) in patients with chronic lung disease referred for lung transplantation.

**Methods:**

A retrospective observational study with patients with pulmonary emphysema or fibrosis referred for transplantation between 2012 and 2016. Pulmonary hypertension was defined as mean pulmonary artery pressure ≥25mmHg, or between 21 and 24mmHg, with pulmonary vascular resistance >3 Wood units on right heart catheterization. Tomographic measurements were made by two independent radiologists.

**Results:**

This sample comprised 13 patients with emphysema and 19 patients with pulmonary fibrosis. Of these, 18 had pulmonary hypertension. The level of agreement in tomographic measurements made by radiologists was high (intraclass correlation coefficients 0.936 and 0.940, for pulmonary artery diameter and pulmonary artery diameter to ascending aorta diameter ratio, respectively). Areas under the ROC curves constructed for pulmonary artery diameter, pulmonary artery diameter to ascending aorta diameter ratio, and pulmonary artery systolic pressure as predictors of pulmonary hypertension were 0.540, 0.629 and 0.783, respectively. The sensitivity, specificity and negative predictive value of pulmonary artery systolic pressure ≥40mmHg were 67%, 79% and 65%, respectively. The combined criterion (pulmonary artery diameter to ascending aorta diameter ratio >1 and/or pulmonary artery systolic pressure ≥40mmHg) achieved sensitivity of 72%, specificity of 79%, and a negative predictive value of 69%.

**Conclusion:**

Measurements of pulmonary artery and ascending aorta diameter were highly reproducible. The association of pulmonary artery and aortic diameter >1 and/or pulmonary artery systolic pressure ≥40mmHg improved the sensitivity and the negative predictive value for pulmonary hypertension screening. This strategy demands prospective validation to assess safety and cost-effectiveness.

## INTRODUCTION

Pulmonary hypertension (PH) may result from chronic parenchymal lung disease and is a marker of disease severity.^( 1 , 2 )^ Pulmonary hypertension affects up to 90% of patients with chronic obstructive pulmonary disease (COPD) GOLD IV, and more than 60% of patients with end-stage idiopathic pulmonary fibrosis.^( 3 )^ Hence, PH is a common comorbidity in patients referred for lung transplantation, with potential implications for perioperative management and negative impacts on post-transplant survival.^( 4 - 6 )^

In this context, screening of patients referred for lung transplantation for PH is vital, not only from an eligibility standpoint, but also for appropriate planning of resources required for intra and postoperative clinical support.^( 7 )^ In the population at large, initial PH assessment is based on transthoracic echocardiography. However, prior studies have disputed the accuracy of this imaging modality in patients with advanced chronic lung disease, who may be particularly difficult to examine due to poor acoustic window.^( 8 )^

Right heart catheterization is routinely used in several lung transplant centers worldwide to screen transplant candidates suffering from interstitial lung disease (ILD) or COPD for PH. This practice is supported by specialists, who recommend right heart catheterization in patients with advanced lung disease whenever the diagnosis may directly interfere with therapeutic planning.^( 3 )^ Indeed, catheterization is the gold standard for PH diagnosis in these individuals.^( 3 )^ However, it is an invasive procedure with limited availability and high costs. Therefore, investigation of alternative, non-invasive and lower cost strategies is welcome.

Prior studies with COPD^( 9 )^ and ILD patients^( 2 , 10 , 11 )^ have tried to determine whether measurements of pulmonary artery diameter (PAd), taken from chest computed tomography (CT) images with no contrast enhancement, could be used for PH diagnosis and prognostic prediction. It has been suggested that PAd >30mm and pulmonary artery to ascending aorta diameter ratio (PAd/Aod) >1 are indicative of PH. However, the accuracy of these measurements varies considerable among publications.^( 2 , 9 - 11 )^ In many of these studies, PH is defined as mean pulmonary artery pressure (mPAP) higher than 25mmHg. This criterion has recently been modified, following the World Symposium on Pulmonary Hypertension. Since 2019, PH is defined as mPAP ≥25mmHg or between 21 and 24mmHg, with evidence of increased pulmonary vascular resistance (PVR >3 Wood units).^( 3 )^

## OBJECTIVE

To examine the accuracy of a pulmonary hypertension screening strategy based on a combination of echocardiographic data and tomographic measurements ( pulmonary artery diameter, and pulmonary artery diameter to ascending aorta diameter ratio) in patients with chronic lung disease referred for lung transplantation.

## METHODS

A retrospective observational study with patients with COPD and/or ILD referred for assessment and potential enrollment in Public Health System Transplant Program ( *Programa de Apoio ao Desenvolvimento Institucional do Sistema Único de Saúde -* PROADI-SUS) of the Brazilian Ministry of Health and *Hospital Israelita Albert Einstein* , between 2012 and 2016. This study was approved by the research ethics committee of *Hospital Israelita Albert Einstein* (HIAE), opinion 3.515.313, CAAE: 09443118.2.0000.0071. Patients with missing CT and/or right heart catheterization data and patients undergoing CT and hemodynamic assessment more than 6 months apart were excluded.

Data collection was based on medical record analysis following approval by the ethics committee, with waiver of informed consent. The following pieces of data were retrieved: demographic data, underlying lung disease, lung function, 6-minute walk test, estimated echocardiographic pulmonary artery systolic pressure (PASP), and right heart catheterization variables of interest (mPAP, PVR and pulmonary capillary pressure).

Echocardiographic assessment and cardiac catheterization were performed by different physicians, as per duty schedules. Echocardiograms revealing right chamber enlargement and/or elevated PASP were considered consistent with PH. In spite of current recommendations (adoption of direct measurements of tricuspid regurgitation velocity rather than PASP estimates),^( 12 )^ this parameter was not routinely recorded in medical reports at the time 2012 to 2016. Therefore, PSAP data were used. Historically, studies investigating PH in patients with chronic lung disease have adopted different PSAP cut-offs (≥36mmHg or ≥40mmHg in most cases).^( 13 - 16 )^ Given the prognostic significance of PH for lung transplantation and the need to avoid false-negative results in screening programs, patients in this sample were first categorized according to the 36mmHg, then to the 40mmHg cut-off. Pulmonary hypertension was defined as mPAP ≥25mmHg, or between 21 and 24mmHg with evidence of PVR >3 Wood units, as per the World Symposium on Pulmonary Hypertension recommendations.^( 3 )^

All chest CT exams were performed without contrast enhancement. Tomographic measurements PAd and Aod were made by two radiology specialists in an independent and blinded fashion. Pulmonary artery diameter was measured at the level of the bifurcation and the largest Aod measured in the corresponding cross-section.^( 17 )^

### Statistical analysis

Convenience sampling was used in this study. The reproducibility of PAd and Aod measurements made by two radiologists was assessed using Bland-Altman plots and intraclass correlation coefficients. Mean PAd and Aod values provided by both radiologists were used in the analysis. Correlations between PAd and mPAP, and between PAd/Aod and mPAP were investigated using the Spearman correlation test. The Mann-Whitney test was used to compare numerical variables between patients with and without PH. Receiver operating characteristic (ROC) curves were constructed to compare the discriminative ability of PAd, PAd/Aod and PASP for PH diagnosis. The sensitivity, specificity, and negative predictive values of PAd, PAd/Aod and PASP (isolated and combined) for PH diagnosis were also calculated. The following cut-offs were adopted: PAd >30mm, PAd/Aod >1 and PASP ≥36mmHg or 40mmHg.

Patients with mPAP between 21 and 24mmHg and no PVR data in medical records were considered to be unaffected by PH, since there is no evidence to support the impact of such mPAP values on post-transplant mortality or surgical procedure selection. Subsequent sensitivity analysis excluding these patients was conducted. Analyses were carried out using software SPSS, version 26. The level of significance was set at p value <0.05.

## RESULTS

### Demographic data and lung function

This sample comprised 32 patients (13 patients with COPD and 19 patients with ILD). Of these, 18 had PH based on cardiac catheterization findings. Demographic, lung function and hemodynamic assessment data are given in table 1 . Resting lung function and 6-minute walk test variables did not differ significantly between patients with and without PH ( Table 2 ).

**Table 1:** Demographic, functional and hemodynamic characteristics of patients with chronic obstructive pulmonary disease or interstitial lung disease assessed for heart transplantation o

Variables	COPD	ILD

	(n=13)	(n=19)
Sex, male:female	4:9	9:10
Age, years	60 (57-63)	57 (50- 59)
FEV_1_, % of predicted	19 (16-29)	47 (32-69)
FVC, % of predicted	55 (48-75)	39 (29-58)
DLCO, % of predicted	25 (22-47)	42 (28-56)
6MWT (m)	265 (335-377)	320 (275-357)
SpO_2_ final 6MWT, %	90 (82-94)	85 (79-87)
PH, yes:no	8:5	10:9
mPAP, mmHg	25 (21-28)	24 (18-38)
PASP, mmHg	37 (29-57)	39 (32-53)

Results expressed as median (interquartile range) whenever not otherwise specified. COPD: chronic obstructive pulmonary disease; ILD: interstitial lung disease; FEV_1_: forced expiratory volume in the first second; FVC: forced vital capacity; DLCO%: carbon monoxide diffusing capacity; 6MWT: distance covered in the 6-minute walk test; SpO_2_: final 6MWT: blood oxygen saturation at the end of the 6-minute walk test; PH: pulmonary hypertension; mPAP: mean pulmonary artery pressure; PASP: pulmonary artery systolic pressure.

**Table 2:** Lung function and 6-minute walk test, according to presence or absence of pulmonary hypertension

Variable	COPD		ILD	
	
	PH (n=5)	No PH (n=8)	PH (n=10)	No PH (n=9)
FEV_1_, % of predicted	18 (15-29)	21 (16-52)	44 (34-56)	50 (25-82)
FVC, % of predicted	55 (50-68)	54 (42-82)	37 (28-46)	44 (30-70)
DLCO, % of predicted	25 (22-25)	25 (22-43)	32 (18-42)	50 (40-82)
6MWT (m)	255 (227-292)	395 (257-540)	330 (230-360)	310 (280-405)
SpO_2_ final, %	91 (88-94)	83 (76-94)	81 (70-87)	86 (82-90)
ΔSpO_2(final – initial)_	-3 (-5-0)	-12 (-14- -3)	-16 (-22- -8)	-9 (-15- -5)

COPD: chronic obstructive pulmonary disease; ILD: interstitial lung disease; PH: pulmonary hypertension; FEV_1_: forced expiratory volume in the first second; FVC: forced vital capacity; DLCO%: carbon monoxide diffusing capacity; 6MWT: distance covered in the 6-minute walk test; SpO_2_: final: blood oxygen saturation at the end of the 6-minute walk test; ΔSpO_2(final – initial)_: variation in blood oxygen saturation at start and end of the 6-minute walking test.

### Reproducibility of pulmonary artery and ascending aorta diameter measurements

**Figure 1:**
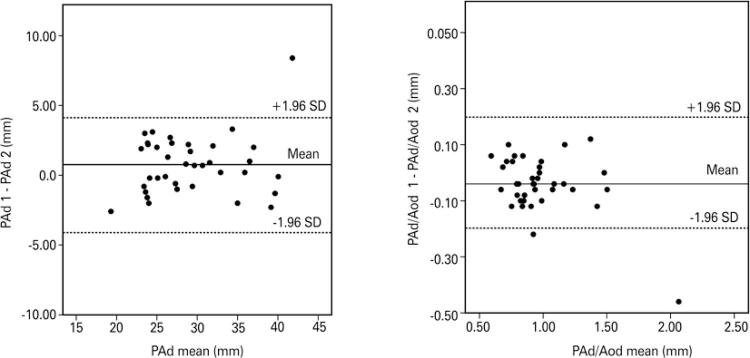
Bland-Altman plots depicting reproducibility indices assigned to computed tomographic measurements (pulmonary artery diameter, and pulmonary artery to ascending aorta diameter ratio) made by two independent radiologists

PAd: pulmonary artery diameter; Aod: aortic diameter; SD: standard deviation.

The level of agreement between CT measurements made by different radiologists was high. Intraclass correlation coefficients were higher than 0.8 in all cases (PAd: 0.936 [0.879-0.967]; PAd/Aod: 0.940 [0.887-0.969]). Differences between measurements were small (absolute values) and differences between means close to zero in both cases ( Figure 1 ).

### Pulmonary artery systolic pressure, pulmonary artery diameter, and pulmonary artery diameter to ascending aorta diameter ratio, as predictors of pulmonary hypertension

No correlations were found between PAd and mPAP or between PAd/Aod and mPAP in this sample (p>0.05). Median PAd (interquartile range) did not differ between patients with and without PH 26.7mm, (23.9-33.2) *versus* 26.9mm (23.9-29.2) respectively; (p=0.79). Likewise, PAd/Aod did not differ between patients with and without PH 0.89 (0.77-1.08) *versus* 0.88 (0.78-0.93) respectively; (p=0.40). Tomographic measurements did not differ between patients with and without PH, even after stratification according to underlying disease.

Pulmonary artery systolic pressure data were missing in echocardiographic reports of 2 out of 32 patients (one with COPD and one with ILD). Given the lack of indirect signs of PH, these patients were assumed to have PASP lower than the established cut-off (36 or 40mmHg depending on the analysis) for classification purposes. Areas under the ROC curves constructed for PAd, PAd/Aod and PASP as independent predictors of PH were 0.540, 0.629 and 0.783, respectively. In spite of 100% specificity and higher Youden index assigned to PAd/Aod, the PAd/Aod >1 and/or PASP of ≥40mmHg criterion provided the best combination of sensitivity and negative predictive value ( Table 3 ).

**Table 3:** Accuracy of different non-invasive pulmonary hypertension screening strategies relative to cardiac catheterization

Variable	Sensitivity	Specificity	NPV	Youden index
PAd >30mm	0.33	0.85	0.5	0.18
PAd/Aod >1	0.72	1	0.54	0.72
PASP ≥36mmHg	0.67	0.57	0.57	0.24
PASP ≥40mmHg	0.67	0.79	0.65	0.46
PAd/Aod >1 and/or PASP ≥36mmHg	0.72	0.57	0.62	0.29
PAd/Aod >1 and/or PASP ≥40mmHg	0.72	0.79	0.69	0.51

NPV: negative predictive value; PAd: pulmonary artery diameter; PAd/Aod: pulmonary artery diameter to ascending aorta diameter ratio; PASP: pulmonary artery systolic pressure.

Overall, eight patients were incorrectly classified following application of the PAd/Aod >1 and/or PASP ≥40mmHg screening criteria. Of these, three were false-positive (PH ruled out following right heart catheterization). In contrast, other patients (two with pulmonary fibrosis and three with COPD) were false-negative (reclassified as having PH following catheterization). Mild PH prevailed in patients with false-negative results in non-invasive screening (mPAP 25mmHg, 27mmHg and 46mmHg, two, two and one patient, respectively).

### Sensitivity analysis

Four patients with mPAP between 21 and 24mmHg and missing PVR data in medical records were not included in sensitivity analysis. These patients had PASP <36 mmHg and PAd/Aod <1. The accuracy of the PAd/Aod >1 and/or PASP ≥40mmHg was significantly affected by exclusion of these individuals. In this second analysis, sensitivity remained unchanged (72%). However, specificity and negative predictive value dropped to 70% and 58%, respectively.

In the overall sample (n=32) as well as in sensitivity analysis, the combined criterion would have changed the classification of a single patient relative to isolated analysis of echocardiographic findings. This patient would be a false negative based on PASP ≥40mmHg, but correctly identified as having PH according to the combined criterion.

## DISCUSSION

In this study, combined application of echocardiographic data and PAd/Aod ratio was more sensitive and had higher negative predictive value for PH than any of these methods alone, in a population of patients with advanced chronic lung disease on the transplant waiting list. In this sample, the combination of PAd/Aod >1 and/or PASP ≥40mmHg was more specific and had a higher negative predictive value for PH diagnosis relative to the PASP 36mmHg cut-off. Patients with and without PH could not be appropriately distinguished based on PAd alone. Still, this study revealed excellent reproducibility of PAd and PAd/Aod measurements made by independent radiologists.

Transthoracic Doppler echocardiogram is the current method of choice for PH screening, regardless of etiology. Nonetheless, the accuracy of this method in patients with chronic lung disease is limited.^( 14 )^ Pulmonary hypertension is one of the eligibility criteria for inclusion in transplant lists,^( 7 )^ and may impact the selection of anesthetic procedures (use of extracorporeal circulation or intraoperative extracorporeal membrane oxygenation) as well as post-transplant survival.^( 18 - 20 )^ Individual mPAP and PCP values may be used as transplant prioritization criteria in countries adopting the lung allocation score (LAS).^( 21 )^ Therefore, these patients are often submitted to right heart catheterization.

However, right heart catheterization is an invasive and expensive procedure. In the United States, average costs covered by Medicare range from US$ 1,359.00 to US$ 2,810.00, depending on the type of facility (outpatient or inpatient). Similar costs apply to Brazilian private health services. Also, despite high levels of safety, catheterization can be not a comfortable examination. Systematic use of this procedure could be justified if there was evidence of cost-effectiveness. But this hypothesis has not been investigated to date. According to Keller et al., LAS scoring is leading to a significant increase in transplant patient severity of illness, with resultant impacts on lung transplantation costs in the United States, since post-transplant mortality is also higher among patients with more severe illness.^( 22 )^

Refinement of candidate selection criteria and rational use of health resources are necessary to restrain the escalation of transplant costs. Given most patients with advanced lung disease are submitted to chest CT for diagnostic/prognostic purposes, investigation of CT variables capable of predicting PH is warranted. High reproducibility of tomographic measurements of PAd and Aod was reported in prior and in this study.^( 2 , 23 )^ Correlations between PAd/Aod and mPAP have not been demonstrated to date.^( 9 , 10 )^ Nevertheless, PAd/Aod is associated with PH and mortality in patients with pulmonary fibrosis or COPD.^( 2 , 9 )^

Sadly, this finding alone does not support the use the method for initial PH screening. Like in other studies, this analysis revealed that PAd/Aod >1 is highly specific for PH diagnosis (100% specificity in this sample). However, low negative predictive value (54%) is a significant limiting factor.^( 9 )^ For this reason, other non-invasive variables are being investigated as potential predictors of PH in patients with chronic lung disease. A composite score combining partial pressure of oxygen (PaO_2_), percentage of %DLCO (%DLCO), and PAd/Aod to screen for mPAP higher than 21mmHg in patients with idiopathic pulmonary fibrosis has recently been proposed by Furukawa et al.,^( 24 )^ Practical application of this score is limited by lack of validation in other patient populations, and the fact patients with more severe illness may not be able to complete maneuvers required to measure carbon monoxide diffusing capacity. For example, %DLCO values were missing in records of 14 patients in this sample. In contrast, tomographic PAd and Aod measurements and echocardiographic data were available in all cases.

Hence, combined application of CT and echocardiographic criteria appears to be the best non-invasive alternative for PH screening. In this study, the presence of PAd/Aod >1 and/or PASP ≥40mmHg significantly increased the negative predictive value 69%, while maintaining 72% sensitivity for PH diagnosis. Use of this screening strategy led to misclassification of eight out of 32 patients (25%). Four out of five false- negative results were diagnosed with mild PH in invasive hemodynamic assessment. Had this screening strategy been used in this patient population, the number of catheterization procedures would have dropped from 32 to 13 and one case of severe PH would have been missed.

The combined criterion performed poorly on sensitivity analysis. However, according to novel criteria, it is unlikely the four patients excluded actually suffered from PH. Moreover, the is no evidence to support significant impacts of mPAP values between 21 and 24mmHg on anesthetic planning or post-transplant survival. On the contrary: in patients with idiopathic pulmonary fibrosis, *e.g.,* only values higher than 30mmHg are thought to impact post-transplant survival.^( 18 )^ Hence, findings of this study are in keeping and complement existing data, since they provide a direct comparison of a combination of methods with traditional screening and the gold standard diagnostic method.

This study has several limitations. Firstly, although the combination of tomographic and echocardiographic criteria translated into a higher negative predictive value for PH diagnosis, small sample size and lack of prospective validation of the combined criterion (PAd/Aod >1 and/or PASP ≥40mmHg) preclude definitive conclusions regarding the safety and cost-effectiveness of this screening strategy in patients with chronic lung disease in the transplant list. Secondly, given the heterogeneity of the sample regarding underlying disease, it is not possible to say whether PAd or PAd/Aod would perform better as screening parameters in specific groups of patients. In any case, findings of this study are consistent with those of prior studies with homogeneous populations. Therefore, it is unlikely sample heterogeneity would have impacted the results presented. Thirdly, patient age and the time interval between CT assessment and right heart catheterization may have interfered with PAd/Aod accuracy. This sample comprised primarily older patients. Age is thought to have negative impacts on the ability of tomographic measurements to accurately predict PH.^( 25 , 26 )^ Also, patients submitted echocardiographic and tomographic assessment and right heart catheterization six months apart were included. Despite the lack of an established, ideal time frame, shorter intervals between assessments are thought to improve the correlation between PAd/Aod and mPAP in patients with idiopathic pulmonary fibrosis.^( 2 )^

Finally, patients with PH in this sample were not classified as pre-capillary, post-capillary or mixed. In patients in transplant waiting lists, therapeutic management may be affected by Group 2 (associated with left heart disease) as well as Group 3 (associated with respiratory disease and/or hypoxia) PH. Therefore, this study set out to examine the accuracy of non-invasive methods for PH screening, regardless of etiology.

## CONCLUSION

Measurements of pulmonary artery diameter and pulmonary artery to ascending aorta diameter ratio were highly reproducible. Application of the criterion combining pulmonary artery to ascending aorta diameter ratio >1, and/or pulmonary artery systolic pressure ≥40mmHg achieved higher sensitivity and specificity and had higher negative predictive value for pulmonary hypertension diagnosis than to any of these non-invasive methods alone. The combined screening strategy presented must be validated in large samples and duly tested for safety and cost-effectiveness. Refinement of non-invasive screening strategies may prevent unnecessary right heart catheterization procedures in patients referred for lung transplantation.
