# Supplementary material for: Accuracy of echocardiography and chest tomography for pulmonary hypertension screening in patients awaiting lung transplantation
Source: Einstein (Sao Paulo). 2021 Dec 15;19:eAO5710. doi: 10.31744/einstein_journal/2021AO5710 (PMC8687646; doi:10.31744/einstein_journal/2021AO5710)
Supplement: Translation

# Translation: Acurácia do ecocardiograma e da tomografia de tórax para rastreamento de hipertensão pulmonar em pacientes aguardando transplante de pulmão

**Authors:** Luiza Helena Degani-Costa, João Paulo de Assis, Pedro Paulo Pisaniello Gonçalves, Fernanda Gushken, Gilberto Szarf, José Eduardo Afonso

**Affiliations:** 1 Brasil Hospital Israelita Albert Einstein, São Paulo, SP, Brasil.

**Keywords:** Hipertensão pulmonar, Transplante, Ecocardiografia, Tomografia computadorizada por raios x, Tórax/diagnóstico por imagem, Cateterismo cardíaco

## Abstract

**Objetivo:**

Avaliar a acurácia de uma estratégia de rastreamento de hipertensão pulmonar baseada na combinação de dados de ecocardiograma com as medidas derivadas da tomografia computadorizada (diâmetro da artéria pulmonar e razão entre diâmetro da artéria pulmonar e diâmetro da aorta ascendente) em pacientes pneumopatas crônicos encaminhados para transplante pulmonar.

**Métodos:**

Estudo observacional retrospectivo realizado com pacientes com enfisema e fibrose pulmonar avaliados para transplante entre 2012 e 2016. Definiu-se hipertensão pulmonar como pressão arterial pulmonar média ≥25mmHg, ou entre 21 a 24mmHg, com resistência vascular pulmonar >3 unidades Wood no cateterismo direito. As medidas tomográficas foram realizadas por dois radiologistas independentes.

**Resultados:**

Foram incluídos 13 pacientes com enfisema e 19 com fibrose pulmonar, sendo 18 com hipertensão pulmonar. Houve alta concordância entre os radiologistas em relação às medidas tomográficas (coeficientes de correlação intraclasse para diâmetro da artéria pulmonar de 0,936 e diâmetro da artéria pulmonar/diâmetro da aorta ascendente de 0,940). As áreas abaixo da curva ROC de diâmetro da artéria pulmonar, diâmetro da artéria pulmonar/diâmetro da aorta ascendente, e pressão sistólica da artéria pulmonar como preditores de hipertensão pulmonar foram 0,540, 0,629 e 0,783, respectivamente. A sensibilidade, especificidade e valor preditivo negativo da pressão de sistólica de artéria pulmonar ≥40mmHg foram 67%, 79% e 65%, respectivamente. O critério combinado de diâmetro da artéria pulmonar/diâmetro da aorta ascendente >1 e/ou pressão sistólica da artéria pulmonar ≥40mmHg mostrou sensibilidade de 72%, especificidade de 79%, e valor preditivo negativo de 69%.

**Conclusão:**

Os diâmetros da artéria pulmonar e da aorta ascendente foram altamente reprodutíveis. A associação entre diâmetro da artéria pulmonar e diâmetro da aorta ascendente >1 e/ou pressão sistólica da artéria pulmonar ≥40mmHg melhorou a sensibilidade e o valor preditivo negativo para rastreamento de hipertensão pulmonar. Essa estratégia requer validação prospectiva para se avaliarem segurança e custo-efetividade.

## INTRODUÇÃO

A hipertensão pulmonar (HP) pode se desenvolver como complicação de doenças pulmonares parenquimatosas crônicas, sendo um marcador de gravidade.^( 1 , 2 )^A HP está presente em até 90% dos pacientes com doença pulmonar obstrutiva crônica (DPOC) GOLD IV, e em mais de 60% dos pacientes com fibrose pulmonar idiopática em estágio terminal.^( 3 )^Assim, a HP é uma comorbidade frequente entre pacientes encaminhados para o transplante pulmonar, o que não apenas gera implicações no manejo perioperatório como também pode se associar a menor sobrevida pós-transplante.^( 4 - 6 )^

Nesse contexto, o rastreamento de HP entre pacientes encaminhados para transplante é fundamental não apenas para avaliar sua elegibilidade, mas também para programar adequadamente os recursos necessários para suporte clínico intra e pós-operatório.^( 7 )^Embora, na população geral, a avaliação inicial para HP seja realizada por meio de ecocardiografia transtorácica, estudos prévios colocaram em dúvida sua acurácia em pacientes com pneumopatias crônicas avançadas, nos quais a janela acústica limitada pode tornar o exame particularmente difícil.^( 8 )^

Assim, diversos centros de transplante pulmonar ao redor do mundo adotam como rotina o rastreamento de HP com cateterismo cardíaco direito de repouso para todos os candidatos com doença pulmonar intersticial (DPI) ou DPOC. Essa prática recebe suporte de especialistas, que recomendam a realização de cateterismo cardíaco direito em paciente com pneumopatias avançadas sempre que o diagnóstico influencie diretamente na programação terapêutica.^( 3 )^De fato, o cateterismo é o exame padrão-ouro para o diagnóstico de HP nesses indivíduos,^( 3 )^mas se trata de um teste invasivo, com disponibilidade limitada e de alto custo. Assim, a busca por outras estratégias não invasivas e de menor custo é bem-vinda.

Estudos prévios em pacientes com DPOC^( 9 )^e DPI^( 2 , 10 , 11 )^buscaram avaliar se o diâmetro da artéria pulmonar (dAP), aferido em tomografia computadorizada (TC) de tórax sem contraste, poderia ser usado para predizer prognóstico e presença de HP. Nesse sentido, dAP >30mm e razão entre dAP e diâmetro da aorta ascendente (dAP/dAo) >1 foram sugeridos como indicativos da presença de HP, porém a acurácia dessas medidas varia significativamente entre os trabalhos.^( 2 , 9 - 11 )^Vale ressaltar, no entanto, que muitos destes estudos definiram HP como uma pressão de artéria pulmonar média (PAPm) acima de 25mmHg, critério que recentemente sofreu modificações após o *World Symposium on Pulmonary Hypertension* . Desde 2019, define-se HP nesses indivíduos como presença de PAPm ≥25mmHg, ou entre 21 e 24mmHg, com evidência de aumento da resistência vascular pulmonar (RVP >3 unidades Wood).^( 3 )^

## OBJETIVO

Avaliar a acurácia de uma estratégia de rastreamento de hipertensão pulmonar baseada na combinação de dados de ecocardiograma com as medidas derivadas da tomografia computadorizada (diâmetro da artéria pulmonar, e razão entre diâmetro da artéria pulmonar e diâmetro da aorta ascendente) em pacientes pneumopatas crônicos encaminhados para transplante pulmonar.

## MÉTODOS

Este foi um estudo observacional retrospectivo, no qual foram incluídos os pacientes com DPOC e/ou DPI encaminhados para avaliação para transplante no programa ligado ao Programa de Apoio ao Desenvolvimento Institucional do Sistema Único de Saúde (PROADI-SUS), do Ministério da Saúde e do Hospital Israelita Albert Einstein, entre 2012 e 2016. O estudo foi aprovação pelo comitê de ética em pesquisa do Hospital Israelita Albert Einstein (HIAE), com o parecer 3.515.313, CAAE: 09443118.2.0000.0071. Foram excluídos os pacientes com TC e/ou cateterismo cardíaco direito não disponíveis em prontuário e aqueles cuja TC tivesse sido realizada com intervalo superior a 6 meses em relação à avaliação hemodinâmica.

A coleta de dados foi realizada por análise de prontuários após aprovação do comitê de ética, sendo dispensada a assinatura de Termo de Consentimento Livre e Esclarecido (TCLE) por parte dos indivíduos incluídos no estudo. Foram registrados dados demográficos, doença pulmonar de base, função pulmonar, teste de caminhada de 6 minutos, pressão sistólica da artéria pulmonar (PSAP) estimada pelo ecocardiograma e variáveis de interesse do cateterismo cardíaco direito (PAPm, RVP e pressão capilar pulmonar).

Ecocardiograma e cateterismo cardíaco foram realizados por diferentes médicos, conforme escala de plantão. Foram considerados como compatíveis com HP os ecocardiogramas que mostravam aumento de câmaras direitas e/ou da PSAP. Embora a recomendação atual seja preferir a medida direta da velocidade de regurgitação tricúspide ao invés de estimativas de PSAP,^( 12 )^essa velocidade não era descrita rotineiramente nos laudos de 2012 a 2016, o que justificou o uso da PSAP. Historicamente, estudos de HP em pacientes com doenças pulmonares crônicas têm utilizado diferentes pontos de corte de PSAP, notadamente PSAP ≥36mmHg ou PSAP ≥40mmHg.^( 13 - 16 )^Tendo em vista a importância prognóstica da HP no contexto do transplante pulmonar e a necessidade de se evitarem falsos-negativos no rastreamento, optou-se por, inicialmente, classificar os pacientes de acordo com o corte de 36mmHg e, posteriormente, repetir a análise utilizando-se o corte de 40mmHg. O diagnóstico de certeza de HP baseou-se na presença de PAPm ≥25mmHg, ou entre 21mmHg e 24mmHg com evidência de aumento da RVP >3 unidades Wood, conforme as recomendações do *World Symposium on Pulmonary Hypertension* .^( 3 )^

Todas as tomografias avaliadas foram realizadas sem contraste. As medidas de dAP e dAo na TC de tórax foram realizadas de maneira independente e cega por dois médicos radiologistas. O dAP foi medido em sua bifurcação, com o maior diâmetro da aorta sendo medido no corte correspondente.^( 17 )^

### Análise estatística

Para este estudo, utilizou-se uma amostra de conveniência. A reprodutibilidade das medidas de dAP e dAo realizadas pelos dois radiologistas foi avaliada pelo gráfico de Bland-Altman e pelo coeficiente de correlação intraclasse. A média das medidas de dAP e dAo avaliadas pelos dois radiologistas foi considerada em todas as análises. O teste de correlação de Spearman foi utilizado para avaliar existência de correlação entre dAP e PAPm, assim como entre dAP/dAo e PAPm. O teste de Mann-Whitney foi utilizado para comparar variáveis numéricas entre os pacientes com e sem HP. Análises por meio da curva característica de operação do receptor (ROC - *receiver operating characteristic* ) foram utilizadas para se comparar a capacidade discriminativa de dAP, dAP/dAo e PSAP para o diagnóstico de HP. Adicionalmente, foram calculados sensibilidade, especificidade, valores preditivos negativos da dAP, dAP/dAo e PSAP (individualmente e combinados) para o diagnóstico da HP, considerando-se os pontos de corte estabelecidos para cada método (dAP >30mm, dAP/dAo >1 e PSAP ≥36mmHg ou ≥40mmHg).

Pacientes com PAPm entre 21 e 24mmHg que não tinham registrados em seus prontuários os valores de RVP foram considerados como não portadores de HP, já que não há evidência de que tais níveis de PAPm impactem em mortalidade pós-transplante ou escolha de técnica cirúrgica. Uma análise de sensibilidade foi posteriormente realizada excluindo-se tais pacientes da amostra. Todas as análises foram realizadas utilizando-se o programa SPSS versão 26, e o valor de p<0,05 foi considerado estatisticamente significante.

## RESULTADOS

### Dados demográficos e função pulmonar

A amostra contou com 32 pacientes, sendo 13 com DPOC e 19 com DPI, dos quais 18 indivíduos apresentavam HP, de acordo com o cateterismo cardíaco. Dados demográficos, função pulmonar e avaliação hemodinâmica são apresentados na tabela 1 . Não houve diferença estatística na função pulmonar de repouso e nem nas variáveis do teste da caminhada de 6 minutos entre pacientes com e sem HP ( Tabela 2 ).

**Tabela 1:** Características demográficas, funcionais e hemodinâmicas dos pacientes com doença pulmonar obstrutiva crônica ou doença pulmonar intersticial avaliados para transplante cardíaco

Variáveis	DPOC	DPI

	(n=13)	(n=19)
Sexo, masculino:feminino	4:9	9:10
Idade, anos	60 (57-63)	57 (50- 59)
VEF_1_, % do previsto	19 (16-29)	47 (32-69)
CVF, % do previsto	55 (48-75)	39 (29-58)
DLCO, % do previsto	25 (22-47)	42 (28-56)
DC6M (m)	265 (335-377)	320 (275-357)
SpO_2_final TC6M, %	90 (82-94)	85 (79-87)
HP, sim:não	8:5	10:9
PAPm, mmHg	25 (21-28)	24 (18-38)
PSAP, mmHg	37 (29-57)	39 (32-53)

Resultados expressos por mediana (intervalo interquartil) quando não identificado de outra forma. DPOC: doença pulmonar obstrutiva crônica; DPI: doença pulmonar intersticial; VEF_1_: volume expiratório forçado no primeiro segundo; CVF: capacidade vital forçada; DLCO%: capacidade de difusão de monóxido de carbono; DC6M: distância caminhada em 6 minutos; SpO_2_final TC6M: saturação de oxigênio no sangue ao final do teste de caminhada de 6 minutos; HP: hipertensão pulmonar; PAPm: pressão arterial pulmonar média; PSAP: pressão sistólica arterial pulmonar.

**Tabela 2:** Função pulmonar e teste de caminhada de 6 minutos, de acordo com a presença ou a ausência de hipertensão pulmonar

Variável	DPOC		DPI	
	
	HP (n=5)	Sem HP (n=8)	HP (n=10)	Sem HP (n=9)
VEF_1_, % do previsto	18 (15-29)	21 (16-52)	44 (34-56)	50 (25-82)
CVF, % do previsto	55 (50-68)	54 (42-82)	37 (28-46)	44 (30-70)
DLCO, % do previsto	25 (22-25)	25 (22-43)	32 (18-42)	50 (40-82)
DC6M, m	255 (227-292)	395 (257-540)	330 (230-360)	310 (280-405)
SpO_2_final, %	91 (88-94)	83 (76-94)	81 (70-87)	86 (82-90)
ΔSpO_2(final – inicial)_	-3 (-5-0)	-12 (-14- -3)	-16 (-22- -8)	-9 (-15- -5)

DPOC: doença pulmonar obstrutiva crônica; DPI: doença pulmonar intersticial; HP: hipertensão pulmonar; VEF_1_: volume expiratório forçado no primeiro segundo; CVF: capacidade vital forçada; DLCO%: capacidade de difusão de monóxido de carbono; DC6M: distância caminhada em 6 minutos; SpO_2_final: saturação de oxigênio no sangue ao final do teste de caminhada de 6 minutos; ΔSpO_2(final – inicial)_: variação da saturação de oxigênio no sangue entre início e fim do teste de caminhada de 6 minutos.

### Reprodutibilidade das medidas de diâmetro da artéria pulmonar e aorta

A concordância das medidas tomográfica entre radiologistas foi alta, com valores de coeficientes de correlação intraclasse invariavelmente acima de 0,8 (dAP: 0,936 [0,879-0,967]; dAP/dAo: 0,940 [0,887-0,969]). A diferença entre as medidas foi pequena em valores absolutos, sendo sua média próxima de zero para as duas variáveis ( Figura 1 ).

**Figura 1:** Gráficos de Bland-Altman demonstrando reprodutibilidade dos índices tomográficos (diâmetro da artéria pulmonar e diâmetro da artéria pulmonar/diâmetro da aorta) medidos por dois radiologistas independentes

dAP: diâmetro da artéria pulmonar; dAo: diâmetro da aorta; DP: desvio padrão.

### Pressão sistólica da artéria pulmonar, diâmetro da artéria pulmonar e diâmetro da artéria pulmonar e dâmetro da aorta ascendente como preditores de hipertensão pulmonar

Não houve correlação entre a dAP e PAPm e nem entre dAP/dAo e PAPm na amostra total (p>0,05). A mediana (intervalo interquartil) da dAP foi semelhante para doentes com e sem HP, com dAP de 26,7mm (23,9-33,2) *versus* 26,9mm (23,9-29,2), respectivamente (p=0,79). Da mesma forma, dAP/dAo também não diferiu entre os grupos, sendo de 0,89 (0,77-1,08) *versus* 0,88 (0,78-0,93) em indivíduos com e sem HP (p=0,40). Não houve diferença nas medidas tomográfica entre pacientes com e sem HP mesmo quando estratificados pela doença de base.

Dos 32 pacientes, apenas dois (um com DPOC e outro com DPI) não tinham o valor de PSAP descritos em seus laudos de ecocardiograma. Nesses dois casos, não exisitam sinais indiretos de HP e, para fins de categorização, ambos foram classificados como tendo PSAP abaixo dos pontos de corte estabelecidos (36 ou 40mmHg, a depender da análise). As áreas abaixo da curva ROC de dAP, dAP/dAo e PSAP como preditores isolados de HP foram 0,540, 0,629 e 0,783, respectivamente. Embora dAP/dAo tenha mostrado especificidade de 100% e o maior índice de Youden, o critério dAP/dAo >1 e/ou PSAP ≥40mmHg permitiu a melhor combinação de sensibilidade e valor preditivo negativo ( Tabela 3 ).

**Tabela 3:** Acurácia das diferentes estratégias de rastreamento não invasivo de hipertensão pulmonar em relação ao cateterismo cardíaco

Variável	Sensibilidade	Especificidade	VPN	Índice de Youden
dAP >30mm	0,33	0,85	0,5	0,18
dAP/dAo >1	0,72	1	0,54	0,72
PSAP ≥36mmHg	0,67	0,57	0,57	0,24
PSAP ≥40mmHg	0,67	0,79	0,65	0,46
dAP/dAo >1 e/ou PSAP ≥36mmHg	0,72	0,57	0,62	0,29
dAP/dAo >1 e/ou PSAP ≥40mmHg	0,72	0,79	0,69	0,51

VPN: valor preditivo negativo; dAP: diâmetro da artéria pulmonar; dAP/dAo: razão entre diâmetro da artéria pulmonar e diâmetro da aorta ascendente; PSAP: pressão sistólica arterial pulmonar.

No total, oito pacientes foram classificados erroneamente quando utilizado o critério de rastreamento dAP/dAo >1 e/ou PSAP ≥40mmHg. Destes, três tiveram a hipótese de HP excluída no cateterismo cardíaco direito (falsos-positivos). Por outro lado, dois pacientes com fibrose pulmonar e três com DPOC foram reclassificados como portadores de HP após o cateterismo (falsos-negativos). Os pacientes falsos-negativos pelo rastreamento não invasivo tiveram predominantemente HP leve (dois pacientes com PAPm de 25mmHg, dois com PAPm de 27mmHg e 1 com PAPm de 46mmHg).

### Análise de sensibilidade

Quatro pacientes com PAPm entre 21 e 24mmHg estavam sem RVP registrada em prontuário e foram excluídos na análise de sensibilidade, e todos apresentavam PSAP <36mmHg e dAP/dAo <1. A exclusão desses indivíduos da amostra alterou significativamente a acurácia do critério combinado dAP/dAo >1 e/ou PSAP ≥40mmHg. Nessa nova análise, a sensibilidade manteve-se em 72%, porém houve queda da especificidade e do valor preditivo negativo para 70% e 58%, respectivamente.

Tanto na amostra total (n=32) quanto na análise de sensibilidade, o critério combinado teria mudado a classificação de apenas um paciente em comparação à análise isolada do ecocardiograma (inicialmente classificado como falso-negativo pela PSAP ≥40mmHg, mas que teria sido corretamente identificado como portador de HP com o critério combinado).

## DISCUSSÃO

Neste estudo, demonstrou-se que a combinação de ecocardiograma com a dAP/dAo teve melhor desempenho em termos de sensibilidade e valor preditivo negativo para HP do que qualquer um dos testes isoladamente, em uma população de paciente com pneumopatias crônicas avançadas em lista de transplante. Nesta amostra, a combinação de dAP/dAo >1 e/ou PSAP ≥40mmHg permitiu maior especificidade e valor preditivo negativo para o diagnóstico de HP do que quando o corte de 36mmHg foi utilizado para a PSAP. Além disso, embora o dAP isoladamente não tenha permitido boa discriminação entre pacientes com e sem HP, demonstrou-se excelente reprodutibilidade de sua medida e da dAP/dAo entre radiologistas independentes.

O ecocardiograma Doppler transtorácico é, atualmente, o método recomendado para a rastreamento de HP, independentemente da causa. Entretanto, sua acurácia em pacientes pneumopatas crônicos é limitada.^( 14 )^A presença de HP é um dos critérios de indicação para inclusão em lista de transplante,^( 7 )^podendo determinar aspectos da técnica anestésico-cirúrgica (utilização de circulação extracorpórea ou oxigenação por membrana extracorpórea no intraoperatório) e impacto na sobrevida pós-transplante.^( 18 - 20 )^Além disso, valores individuais de PAPm e PCP podem ser utilizados para priorização na lista de transplante em países que utilizam o *lung allocation score* (LAS).^( 21 )^Por esses motivos, frequentemente se opta por submeter todos os pacientes ao cateterismo cardíaco direito.

O cateterismo direito, porém, é um exame invasivo e caro. Nos Estados Unidos, seu preço médio pago pelo *Medicare* varia de US$ 1,359.00 a US$ 2,810.00 dependendo do local onde ele é realizado (centros cirúrgicos ambulatoriais ou hospitais). No Brasil, por sua vez, os valores praticados em serviços de saúde privados não são muito diferentes disso. Além disso, embora seguro, o cateterismo pode ser um exame desconfortável. Tudo isso seria justificável se houvesse evidência de que sua realização sistemática é custo-efetiva. No entanto, além de nenhum estudo ter testado especificamente essa hipótese, Keller et al., demonstraram que o uso do LAS vem aumentando significativamente a gravidade dos pacientes transplantados e, consequentemente, os custos do transplante pulmonar nos Estados Unidos, sendo que a mortalidade pós-transplante é também maior nos pacientes mais graves.^( 22 )^

A contenção da escalada dos custos do transplante passa não apenas pela melhora dos critérios de seleção dos candidatos, mas também pelo uso racional dos recursos de saúde. Considerando que a maior parte dos pacientes com pneumopatias avançadas é submetida a TC de tórax com objetivo diagnóstico e/ou prognóstico, a procura por variáveis tomográficas preditoras de HP se torna lógica. Em estudos prévios e no presente trabalho, a reprodutibilidade das medidas tomográficas de dAP e dAo mostrou-se alta.^( 2 , 23 )^Ademais, embora a existência de boa correlação entre dAP/dAo e PAPm não tenha sido demonstrada neste estudo ou nem em outros,^( 9 , 10 )^Razão em diâmetro da artéria pulmonar e diâmetro da aorta ascendente se associa com a presença de HP e mortalidade tanto em pacientes com fibrose pulmonar quanto com DPOC.^( 2 , 9 )^

Infelizmente, isso, por si só, não torna o método aceitável como investigação inicial de HP. Assim como em outros estudos, demonstrou-se que dAP/dAo >1 apresenta alta especificidade para o diagnóstico de HP (100% nesta amostra), mas o baixo valor preditivo negativo (54%) representa importante limitação.^( 9 )^Por esse motivo, ao longo dos anos, outras variáveis não invasivas vêm sendo estudadas como preditores de HP em pneumopatas crônicos. Recentemente, Furukawa et al. propuseram um escore composto por pressão parcial de oxigênio (PaO_2_), percentagem de difusão de monóxido de carbono (%DLCO), e dAP/dAo para rastreamento de PAPm acima de 21mmHg em paciente com fibrose pulmonar idiopática.^( 24 )^Sua aplicação na prática clínica, entretanto, é comprometida pela ausência de validação em outras populações de pacientes, e pela dificuldade dos pacientes mais graves em realizarem as manobras necessárias à medida da capacidade de %DLCO. Nesta amostra, por exemplo, 14 pacientes não dispunham de valores de %DLCO; por outro lado, a medida de dAP e dAo pela tomografia e a avaliação ecocardiográfica foram possíveis em todos os pacientes.

Assim, a combinação de critérios tomográficos e ecocardiográficos ainda parece ser a melhor forma de rastreamento não invasivo de HP. Neste estudo, a presença de dAP/dAo >1 e/ou PSAP ≥40mmHg permitiu aumentar significativamente o valor preditivo negativo para 69%, enquanto manteve em 72% a sensibilidade para o diagnóstico de HP. Utilizando-se essa estratégia, oito dos 32 pacientes (25%) foram classificados de forma incorreta, sendo que quatro entre os cinco falsos-negativos apresentavam HP leve na avaliação hemodinâmica invasiva. Dessa forma, caso tal estratégia de rastreio tivesse sido utilizada nesta população, o número de cateterismos realizados teria sido reduzido de 32 para 13, mas não teria sido identificado um caso de HP não leve.

Na análise de sensibilidade, o desempenho do critério combinado esteve bem aquém do desejado. Entretanto, é improvável que todos os quatro pacientes excluídos tivessem, de fato, HP de acordo com os novos critérios. Além disso, não há evidências de que valores de PAPm entre 21 e 24mmHg impactem na programação anestésico-cirúrgica ou na sobrevida após o transplante. Pelo contrário: em pacientes com fibrose pulmonar idiopática, por exemplo, apenas valores acima de 30mmHg parecem influenciar na sobrevida pós-transplante.^( 18 )^Dessa forma, os achados deste estudo corroboram a literatura e a expandem, na medida em que se avalia uma combinação de métodos comparados diretamente com o rastreamento tradicional e com o método de diagnóstico padrão-ouro.

Este estudo apresenta várias limitações. Em primeiro lugar, embora a adição do critério tomográfico aos critérios ecocardiográficos tenha aumentado o valor preditivo negativo para o diagnóstico de HP, o pequeno tamanho amostral e a ausência de uma validação prospectiva do critério combinado (dAP/dAo >1 e/ou PSAP ≥40mmHg) não permitem que se chegue a uma conclusão definitiva sobre a segurança e o custo-efetividade dessa estratégia para o rastreamento de HP em pneumopatas crônicos em lista de transplante. Em segundo lugar, a amostra heterogênea em relação à doença de base não permite dizer se, em grupos específicos, dAP ou dAP/dAo teriam melhor desempenho como medidas de rastreio. De qualquer forma, estes achados são comparáveis aos de outros estudos realizados em populações mais homogêneas, sendo, portanto, improvável que esse fator tenha sido determinante para os presentes resultados. Em terceiro lugar, a acurácia de dAP/dAo pode ter sido prejudicada tanto pela idade dos participantes quanto pelo intervalo entre a tomografia e a realização do cateterismo cardíaco direito. A maioria dos pacientes era idosa, porém, a acurácia das medidas tomográficas para predizer HP parece ser negativamente influenciada pela idade.^( 25 , 26 )^Adicionalmente, aceitou-se que os pacientes incluídos na análise tivessem realizado ecocardiograma, tomografia e cateterismo direito num intervalo de até 6 meses. Embora não haja estudo estabelecendo um intervalo ideal, há indícios de que intervalos mais curtos entre os exames melhoram a correlação entre dAP/dAo e PAPm em pacientes com fibrose pulmonar idiopática.^( 2 )^

Por fim, neste estudo, os pacientes com HP não foram classificados em pré-capilares, pós-capilares ou mistos. Entretanto, em pacientes em fila de transplante, tanto a presença de HP Grupo 2 (associada com doença cardíaca esquerda) quanto a do Grupo 3 (associada com doença respiratória e/ou hipoxemia) poderiam potencialmente influenciar na conduta terapêutica, de maneira que o foco deste estudo era avaliar a acurácia dos testes não invasivos para o rastreamento de HP, independentemente de sua etiologia.

## CONCLUSÃO

As medidas de diâmetro da artéria pulmonar e diâmetro da artéria pulmonar/diâmetro da aorta ascendente foram altamente reprodutíveis, e o uso do critério combinado entre diâmetro da artéria pulmonar/diâmetro da aorta ascendente >1, e/ou pressão sistólica da artéria pulmonar ≥40mmHg permitiu a melhor associação de sensibilidade, especificidade e valor preditivo negativo para hipertensão pulmonar quando comparado a qualquer um dos testes não invasivos isoladamente. Essa estratégia combinada precisa ser validada em amostras maiores e, fundamentalmente, testada em relação à segurança e ao custo-efetividade. O aperfeiçoamento de estratégias de rastreamento não invasivo poderia evitar a realização desnecessária de cateterismo cardíaco direito em pacientes avaliados para transplante pulmonar.
